# A Sustainable Approach for Synthesizing (*R*)-4-Aminopentanoic Acid From Levulinic Acid Catalyzed by Structure-Guided Tailored Glutamate Dehydrogenase

**DOI:** 10.3389/fbioe.2021.770302

**Published:** 2022-01-10

**Authors:** Feng Zhou, Yan Xu, Xiaoqing Mu, Yao Nie

**Affiliations:** ^1^ Lab of Brewing Microbiology and Applied Enzymology, School of Biotechnology, Jiangnan University, Wuxi, China; ^2^ Institute of Industrial Technology, Suqian Jiangnan University, Suqian, China

**Keywords:** levulinic acid, reductive amination, glutamate dehydrogenase, structureguided protein engineering, (*R*)-4-aminopentanoic acid

## Abstract

In this study, a novel enzymatic approach to transform levulinic acid (LA), which can be obtained from biomass, into value-added (*R*)-4-aminopentanoic acid using an engineered glutamate dehydrogenase from *Escherichia coli* (*Ec*GDH) was developed. Through crystal structure comparison, two residues (K116 and N348), especially residue 116, were identified to affect the substrate specificity of *Ec*GDH. After targeted saturation mutagenesis, the mutant *Ec*GDH^K116C^, which was active toward LA, was identified. Screening of the two-site combinatorial saturation mutagenesis library with *Ec*GDH^K116C^ as positive control, the *k*_cat_/*K*_m_ of the obtained *Ec*GDH^K116Q/N348M^ for LA and NADPH were 42.0- and 7.9-fold higher, respectively, than that of *Ec*GDH^K116C^. A molecular docking investigation was conducted to explain the catalytic activity of the mutants and stereoconfiguration of the product. Coupled with formate dehydrogenase, *Ec*GDH^K116Q/N348M^ was found to be able to convert 0.4 M LA by more than 97% in 11 h, generating (*R*)-4-aminopentanoic acid with >99% enantiomeric excess (*ee*). This dual-enzyme system used sustainable raw materials to synthesize (*R*)-4-aminopentanoic acid with high atom utilization as it utilizes cheap ammonia as the amino donor, and the inorganic carbonate is the sole by-product.

## Introduction

**FIGURE 1:**
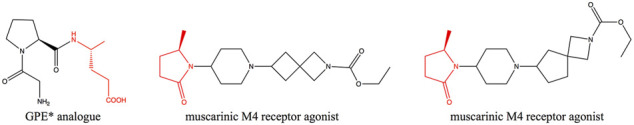
Biologically active molecules containing (*R*)-4-aminopentanoic acid moiety.

Chiral γ-amino acids have attracted increasing attention for their usefulness as building blocks in the pharmaceutical industry and peptide chemistry (Vasudev et al., 2011; Gómez et al., 2017). γ-amino acid scaffolds involved in the synthesis of the peptidomimetics increase the *in vivo* stability and the diversity of peptide molecules (Comegna et al., 2015). Moreover, many peptides containing γ-amino acids have biological activity (Kato et al., 1986; Pettit et al., 1987; Stratmann et al., 1994; Chen et al., 2014). (*R*)-4-aminopentanoic acid is a γ-amino acid with high added-value, which is an important intermediate for the synthesis of Gly-Pro-Glu-OH (GPE*) analogue—a novel class of pharmaceutical agents for the treatment of central nervous system injuries and neurodegenerative diseases including Alzheimer’s, Parkinson’s, and Huntington’s diseases (Trotter et al., 2005)—and muscarinic M_4_ receptor agonist (Brown et al., 2018) (Figure 1), and it can also participate in the formation of physiologically active artificial peptides (Grison et al., 2016). Therefore, the efficient synthesis of (*R*)-4-aminopentanoic acid has become a research hotspot.

**FIGURE 2:**
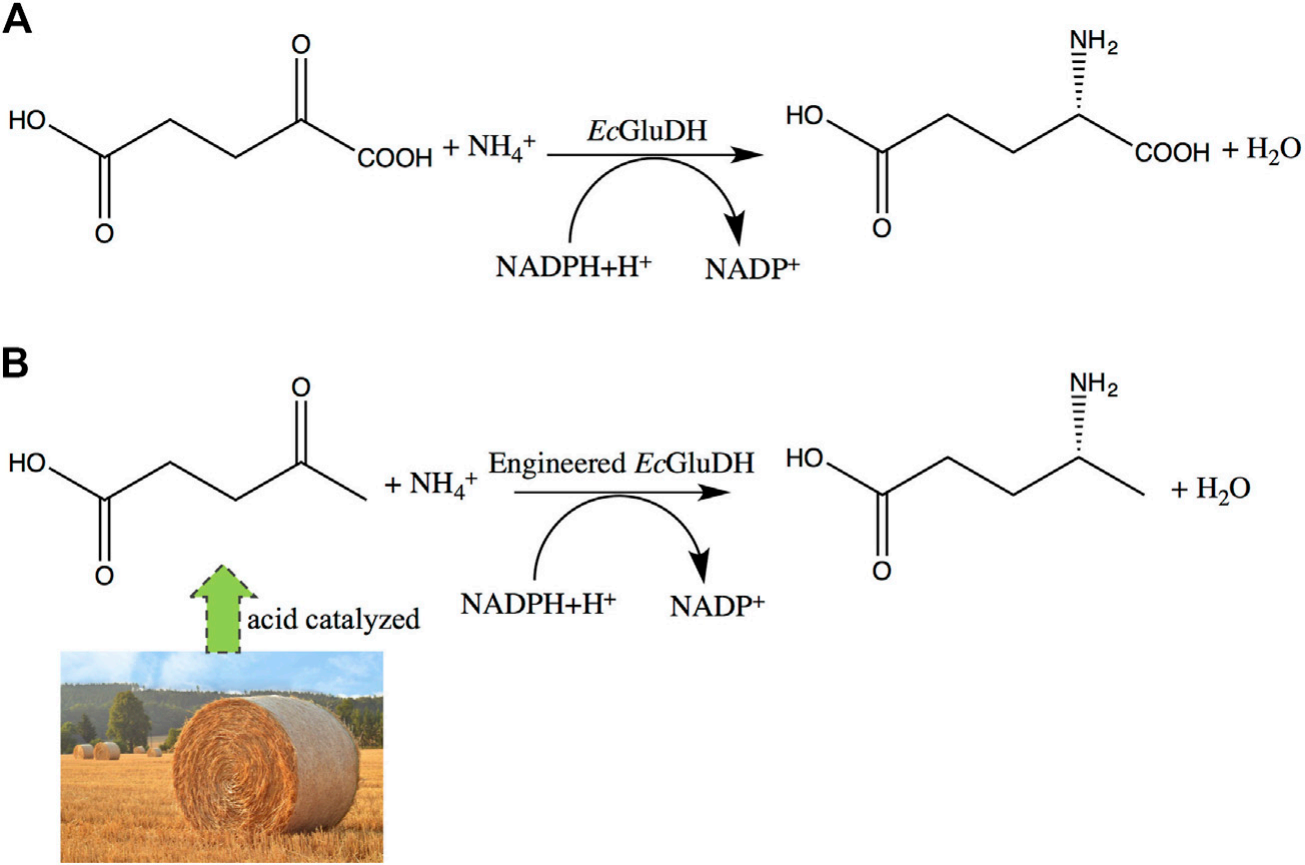
Expected changes in substrate specificity of GDH. **(A)** Wild-type GDH reaction. **(B)** engineered GDH reductive amination of LA to (*R*)-4-aminopentanoic acid.

Levulinic acid (LA) is a promising platform chemical that can be obtained from biomass (Weingarten et al., 2012; Pileidis and Titirici, 2016; Girisuta and Heeres, 2017; Habe et al., 2020; Wang et al., 2020). The synthesis of (*R*)-4-aminopentanoic acid by reductive amination of LA is an attractive reaction route for its sustainable characteristics. The chemical synthesis from LA to (*R*)-4-aminopentanoic acid suffers from poor stereoselectivity (Du et al., 2011; Wu et al., 2019; Xie et al., 2019). Compared with the chemical synthesis of (*R*)-4-aminopentanoic acid, enzymatic methods are environmentally friendly and highly enantioselective. (*R*)-selective amine transaminases can catalyze the synthesis of (*R*)-4-aminopentanoic acid with high optical purity (>99% *ee*) (Jiang et al., 2015); but the reductive amination of carbonyl compounds by transaminases requires organic amine as an amino donor and strategies for shifting the unfavorable thermodynamic equilibrium (Knaus et al., 2017). The reductive amination of LA directly by dehydrogenase to obtain the product is an ideal enzymatic reaction route for its environmentally friendly and high atom economy. Mayol et al. (2016) identified a wild-type amine dehydrogenase from *Petrotoga mobilis* (*Pm*AmDH), which is capable of reductive amination of LA to (*S*)-4-aminopentanoic acid. Subsequently, Cai et al. (2020) engineered *Pm*AmDH through directed evolution and obtained mutants with increased activity, thereby achieving efficient synthesis of (*S*)-4-aminopentanoic acid. However, there are still no relevant reports on the dehydrogenase that converts LA into (*R*)-4-aminopentanoic acid. Engineered amine dehydrogenases modified based on leucine and phenylalanine dehydrogenases are promising enzymes for asymmetric synthesis of (*R*)-chiral amines, as they can use cheap ammonia as amino donor and generate only water as byproduct (Abrahamson et al., 2012; Ye et al., 2015; Patil et al., 2018); but their substrate scopes are restricted to carbonyl compounds without a carboxyl group. Nevertheless, these findings provide motivation for engineering of glutamate dehydrogenase (GDH) for the synthesis of γ-amino acids from LA (Figure 2).

In this study, a combinatorial saturation mutagenesis library on the key sites, which were determined by crystal structure analysis, of GDH from *Escherichia coli* (*Ec*GDH) was constructed and screened, and the best engineered *Ec*GDH^K116Q/N348M^ was obtained. This engineered enzyme can use LA as substrate to efficiently synthesize (*R*)-4-aminopentanoic acid. Hence, this study expands the synthetic scope of amine dehydrogenases and the toolbox of enzymes involved in the synthesis of γ-amino acids.

## Materials and Methods

### Strains, Vectors, and Chemicals

Glutamate dehydrogenase from *E. coli*-K12 (*Ec*GDH, GenBank no. CP047127.1) and formate dehydrogenase of *Burkholderia stabilis* (*Bs*FDH, GenBank no. ACF35003.1) were synthesized by Sangon Biotech (Shanghai, China). The ClonExpress II One Step Cloning Kit were purchased from Vazyme Biotech Company, Ltd. (Nanjing, China). The primers were synthesized by Sangon Biotech (Shanghai, China). Luria-Bertani (LB) media was used for the growth of *E. coli*. PCR reagents were purchased from TaKaRa. The Plasmid Mini Kit I (100), BioSpin PCR Purification Kit, and DNA Gel Extraction Kit (100) were purchased from Omega. LA was supplied by Shanghai Macklin Biochemical Co., Ltd. (Shanghai, China). Racemic 4-aminopentanoic acid was purchased from Taizhou Runsun Chemical Co., Ltd. (Zhejiang, China). All other chemicals were analytical grade and commercially available.

### Construction of Mutagenesis Library

In order to obtain better mutants, combinatorial saturation mutagenesis is selected to combine the sites confirmed by structural analysis. The primers used in this study are shown in Supplementary Table S1. The plasmid pET28a-*Ec*GDH was used as the template. The screening volume of the clones reached 95% library coverage (Reetz et al., 2008).

### High-Throughput Screening

Single colonies created by combinatorial saturation mutagenesis were picked into 96-well microtiter plates containing 1 ml of LB media with 50 μg/ml kanamycin using a QPix420 colony picker. In each plate, four wells were reserved as the controls, two of which are positive controls and the other two are negative controls. The plates were shaken at 37°C for 10 h, then shifted 50 μL of culture to 48-well microtiter plates containing 2 ml of LB media with 50 μg/ml kanamycin for an additional 2.5 h at 37°C, and finally induced with 0.15 mM isopropyl β-D-1-thiogalactopyranoside (IPTG). After 12 h of induction at 17°C, cells were harvested by centrifugation (12857 × *g*, 5 min, 4°C), resuspended in 200 μL lysis buffer (100 mM Tris-HCl buffer, 4 mg/ml lysozyme, pH 7.5), and disrupted by freezing at −80°C for 30 min and by heat shocking at 45°C for 3 min, and then incubated for 10 min at 37°C. After centrifugation (3,724 ×‬ *g*, 20 min, 4°C), the crude enzyme extracts were used for downstream enzymatic reactions.‬‬‬‬‬‬‬‬‬‬‬‬‬‬‬‬‬‬‬‬‬‬‬‬‬‬‬‬‬‬‬‬‬‬‬‬‬‬‬‬‬‬‬‬‬‬‬‬‬‬‬‬‬‬‬‬‬‬‬‬

Combinatorial saturation mutagenesis library was screened using an NADPH auto fluorescence assay (Abrahamson et al., 2012). The assay involved reading the well’s absorbance at two wavelengths, 340 and 600 nm. The decreased absorbance at 340 nm corresponds to the consumption of NADPH, while the 600 nm reading estimates the biomass present in the well. Differences over background in absorbance at 340 nm are normalized by the 600 nm absorbance readings. The change in absorbance at 340 nm for 1 minute was divided by absorbance at 600 nm. The mutants with higher absorbance than wild-type enzyme were selected. The reaction mixture (200 μL) contained 20 μL of crude enzyme extracts, 0.3 mM NADPH, 40 mM LA, 0.8 M NH_4_Cl and 0.1 M Tris-HCl buffer (pH 8.5).

### Expression and Purification of Enzymes

The wild-type and mutants were chosen for purification. Cells were grown in LB medium at 37°C until the OD_600_ reached about 0.6, and then they were induced by 0.1 mM IPTG. The culture was collected by centrifugation and washed three times using normal saline. Then, cell breaking was performed with an ultrasonic cell disruption system (SCIENTZ-IID). After centrifugation at 12857 × *g* for 30 min at 4°C, the clarified supernatant was purified by Ni^2+^-NTA chromatography. The molecular mass of the purified GDH was examined by SDS-PAGE (Schägger, 2006).

### Enzyme Activity Assay

The activity of *Ec*GDH and its mutants was determined at 30°C by monitoring the absorbance change at 340 nm which was corresponding to the concentration variation of NADPH (Li et al., 2014). For reductive amination, the reaction mixture (200 μL) contained 80 mM LA, 0.2 mM NADPH, 0.8 M NH_4_Cl, Tris-HCl buffer (100 mM, pH 8.5), and 20 μL purified enzyme. One unit of enzyme activity was defined as the amount of enzyme catalyzing the reduction of 1 μmol substrate or oxidation of 1 μmol production per minute.

### Kinetic Parameters Determination

Kinetic parameters of the mutants were determined in Tris-HCl buffer (100 mM, pH 8.5) at 30°C with varied concentration of substrate (with concentration range from 80 to 1,600 mM) or NADPH (with concentration range from 0.05 to 0.8 mM). The Michaelis-Menten constants (*K*_m_ and *k*_cat_) were calculated using the nonlinear curve in Origin 8.5.3.

### Synthesis of (*R*)-4-Aminopentanoic Acid by *Ec*GDH^K116Q/N348M^

To compare the reaction performances of *Ec*GDH^K116Q/N348M^ under different conditions (NADP^+^ concentration, pH and temperature), a series of experiments are carried out. In the experiment of the effect of NADP^+^ (with concentration range from 0.25 to 1 mM) on the reaction, 2 ml of reaction mixture contains 0.8 M NH_4_COOH, 100 mM Tris-HCl buffer (pH 8), 80 mM LA, 0.5 mg/ml purified mutant *Ec*GDH^K116Q/N348M^, and 0.03 mg/ml purified *Bs*FDH at 30°C. In the experiment of the effect of pH (pH 7, 8, and 9) on the reaction, 2 ml of reaction mixture contains 0.8 M NH_4_COOH, 100 mM Tris-HCl buffer, 80 mM LA, 0.5 mM NADP^+^, 0.5 mg/ml purified mutant *Ec*GDH^K116Q/N348M^, and 0.03 mg/ml purified *Bs*FDH at 30°C. In the experiment of the effect of temperature (30, 35, 40, and 45°C) on the reaction, 2 ml of reaction mixture contains 0.8 M NH_4_COOH, 100 mM Tris-HCl buffer, 80 mM LA, 1 mM NADP^+^, 0.5 mg/ml purified mutant *Ec*GDH^K116Q/N348M^, and 0.03 mg/ml purified *Bs*FDH. Under the optimal conditions (1 mM NADP^+^, pH 8 and 45°C), the mutant *Ec*GDH^K116Q/N348M^ was tested in 10 ml of reaction mixture, containing 3.2 M NH_4_COOH, 200 mM Tris-HCl buffer, 400 mM LA, 1.51 mg/ml purified mutant *Ec*GDH^K116Q/N348M^, and 0.20 mg/ml purified *Bs*FDH.

### Modeling and Docking

The structures *Ec*GDH^K116C^ and *Ec*GDH^K116Q/N348M^ were modeled using SWISS-MODEL (Schwede et al., 2003). The X-ray structure of *Ec*GDH (PDB: 4BHT) was used as the structural template. The 3D structures of 2-ketoglutarate and LA were obtained by using ChemDraw Ultra. The docking study was processed in AutoDockTool. The wild-type GDH protein was defined as the receptor and 2-ketoglutarate or LA as the ligand, Lys126 was selected as the Flexible Residue, and a box containing Lys92, Ser380, Lys126, Lys116 and Asn348 was set. (The active sites were confirmed by multiple sequence alignments with 5IJZ, 4XGI and 1BGV) And *Ec*GDH^K116C^ or *Ec*GDH^K116Q/N348M^ protein was defined as the receptor and LA as the ligand, Lys126 was selected as the Flexible Residue, and a box containing Lys92, Ser380 and Lys126 was set. Afterward, the docking was automatically processed by running Autogrid and AutoDock in AutoDockTool. After AutoDock running, 10 ligand conformations were generated and their corresponding binding energies were calculated. The best docking model was screened among the 10 docking poses according to the ranks of the models and hydrogen bond formation determined by structure alignment. Both the structures and docking were visualized with PyMOL.

### Analytical Methods

The conversion of LA was determined using HPLC with an Aminex HPX-87H column (300 × 7.8 mm). With the column temperature maintained at 30°C, mobile phase H_2_SO_4_ (5 mM) ran at a flow rate of 0.6 ml/min. (Cai et al., 2020). (*R*)-4-aminopentanoic acid in the reaction mixtures were labeled using 1-fluoro-2,4-dinitrophenyl-5-L-alanineamide (FDAA) and then analyzed on a Develosil ODS-UG-5 column (150 × 4.6 mm). A 10 μL sample of the amino acid, 8 μL of 1 M NaHCO_3_, and 40 μL of 1% (w/v) FDAA in acetone were mixed and heated for 1 h at 40°C. When the sample was cooled to room temperature, 8 μL of 1 N HCl and 934 ml of 40% (v/v) aqueous acetonitrile were added to the mixture, after which it was vortexed and filtered (0.22 mm) for HPLC (Hanson et al., 2008). The HPLC conditions were: mobile phase A 5% acetonitrile (0.05% trifluoroacetic acid, 1% methanol), mobile phase B 60% acetonitrile (0.05% trifluoroacetic acid, 1% methanol), linear gradient from 0% B to 100% B over 45 min at a flow rate 1 ml/min and detected at 340 nm, injection volume 20 μL (Bhushan and Bruckner, 2004; Zhang et al., 2019).

## Results and Discussion

### Structural-Guided Identification of Key Residues at GDH Binding Pocket

**FIGURE 3:**
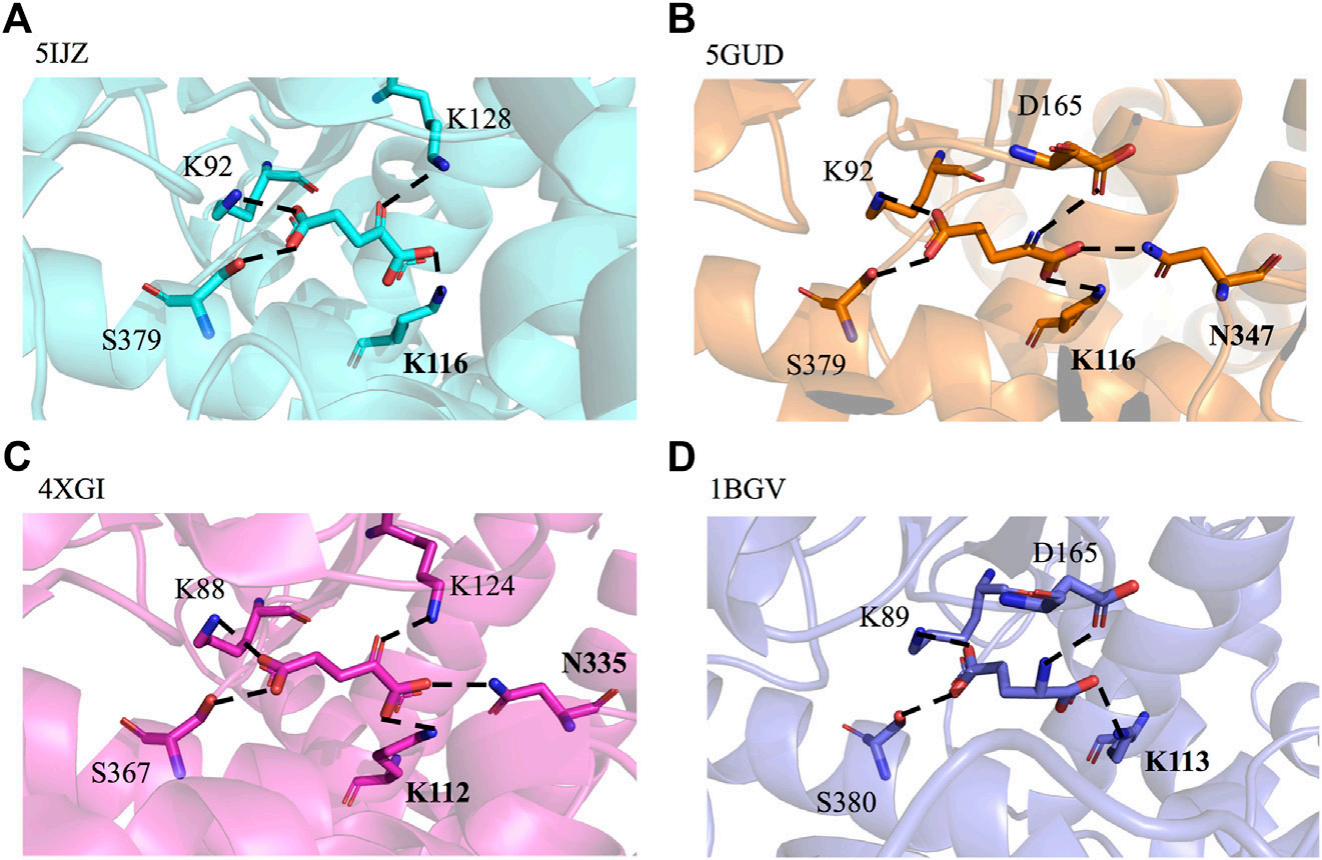
GDH active sites with bound ligand. **(A)** GDH from *Corynebacterium glutamicum* (*Cg*GDH, PDB: 5IJZ), **(B)** GDH from *Corynebacterium glutamicum* (*Cg*GDH, PDB: 5GUD), **(C)** GDH from *Burkholderia thailandensis* (*Bt*GDH, PDB: 4XGI), and **(D)** GDH from *Clostridium symbiosum* (*Cs*GDH, PDB: 1BGV). (the boldface indicates the residues that interact with the main chain carboxyl group of the ligand).

**FIGURE 4:**
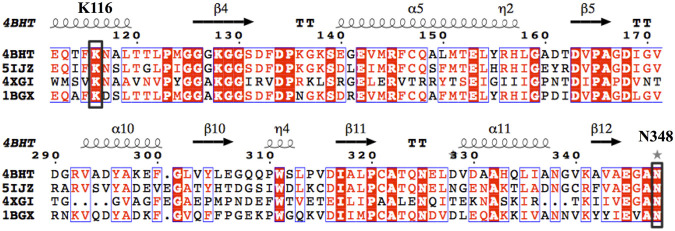
Multiple-sequence alignment of the four selected GDHs as the template. (The sequences were aligned using ClustalO and were presented using ESPript3.0. The two conserved amino acid sites K116 and N348 (numbering according to *Ec*GDH) were indicated by black box.).

By querying the Protein Data Bank database, a large number of crystal structures of GDH were found, including 1BGV (Stillman et al., 1993), 4XGI, 5IJZ, 5GUD (Son et al., 2015) and 4BHT (this study). Unfortunately, there was no ligand in 4BHT. Therefore, the structure of 4BHT was compared with the other four crystal structures (enzyme–ligand complex) to identify the key residues that bind to the ligand. These structures were highly similar to 4BHT, and the root-mean-square deviation (RMSD) was between 0.904 and 2.442. As shown in Figure 3, lysine residues formed hydrogen bonds with the main chain carboxyl group of the ligand in four crystal structures, and additional asparagine residues of 5GUD and 4XGI formed hydrogen bonds with the main chain carboxyl group of the ligand. Therefore, two sites (lysine and asparagine residues), especially lysine residue, were found to play an important role in the binding of the carboxyl group of the ligand backbone. Subsequently, these protein sequences were aligned (Sievers et al., 2011) (Robert and Gouet, 2014), further revealing that these two sites interacted with the main chain carboxyl group are conserved within these enzymes (Figure 4). Therefore, K116 and N348 residues of *Ec*GDH were selected as important sites for targeted mutation.

### Construction and Screening of Mutants With Catalytic Activity for LA

To investigate the effect of K116 on substrate specificity, this residue was mutated into the other 19 amino acids to determine the specific activity toward LA. As shown in Supplementary Table S2, seven mutants (A, C, M, Q, R, S, and T) were found to be active to LA, of which *Ec*GDH^K116C^ had the highest activity (10.1 mU/mg). To obtain mutants with higher activity toward LA, a combinatorial saturation mutagenesis library of residues K116 and N348 was constructed, and mutant *Ec*GDH^K116C^ was used as positive control for screening. To shorten the screening process, NNK degenerate codons were not selected for the saturation mutagenesis. Instead, based on 20 plasmids with different codons at site 116, residue 348 was mutated by four degenerate codons of AHN, TKB, CAT, and CCA encoding all 20 amino acids without introducing redundancy; in each case, 60 transformants were screened for 95% library coverage (Patrick and Firth, 2005; Tang et al., 2012). Thus, the screening amount of transformants could be downscaled from 3,066 to 1,200. (Supplementary Figure S1) Finally, the best mutant *Ec*GDH^K116Q/N348M^ was obtained, and its specific activity (108.6 mU/mg) was determined to be 10.8-times higher than that of *Ec*GDH^K116C^.

**FIGURE 5:**
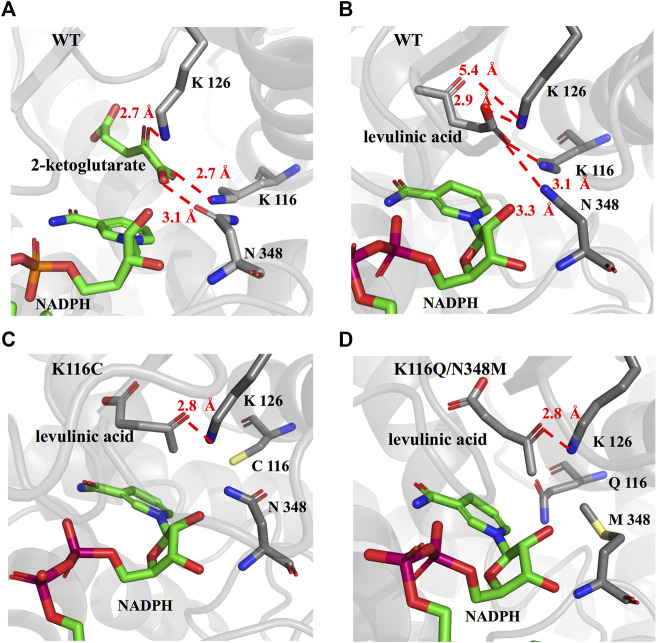
Enzyme-substrate binding pose analysis. **(A)** Enzyme-substrate binding pose for wild type *Ec*GDH with 2-ketoglutarate. **(B)** Enzyme-substrate binding pose for wild type *Ec*GDH with LA. **(C)** Enzyme-substrate binding pose for *Ec*GDH^K116C^ with LA. **(D)** Enzyme-substrate binding pose for *Ec*GDH^K116Q/N348M^ with LA. The distances (in angstrom) are represented by the red dashed lines.

To further explore the role of the *Ec*GDH mutants in the acceptance of LA, the wild-type and mutants (K116C, K116Q/N348M) structure models were used for docking analysis with LA. Figure 5A shows the docking results for wild-type *Ec*GDH and 2-ketoglutarate. The main chain carboxyl group of the ligand was found to interact with K116 and N348, and the carbonyl group of the ligand formed a hydrogen bond with the key catalytic residue K126 (with a distance of 2.7 Å). As shown in Figure 5B, when the ligand was changed from 2-ketoglutarate to LA, the carboxyl group of LA interacted with K116, K126, and N348, which caused catalytically unfavorable poses. With K126 occupied by the carboxyl group, the carbonyl group of LA cannot interact with K126, a residue that is responsible for the formation of an imine intermediate based on the reported mechanism of GDH from the natural substrate (Stillman et al., 1993; Son et al., 2015). This may explain why wild-type *Ec*GDH has no catalytic activity for LA. As shown in Figures 5C,D, after the K116 was modified, the LA showed similar pose to that of 2-ketoglutarate in *Ec*GDH, with a distance of 2.8 Å between the substrate carbonyl-O atom and K126 owing to a hydrogen bond. Therefore, *Ec*GDH^K116C^ and *Ec*GDH^K116Q/N348M^ have catalytic activity toward LA.

### Stereoconfiguration of the Products Catalyzed by *Ec*GDH^K116Q/N348M^

**FIGURE 6:**
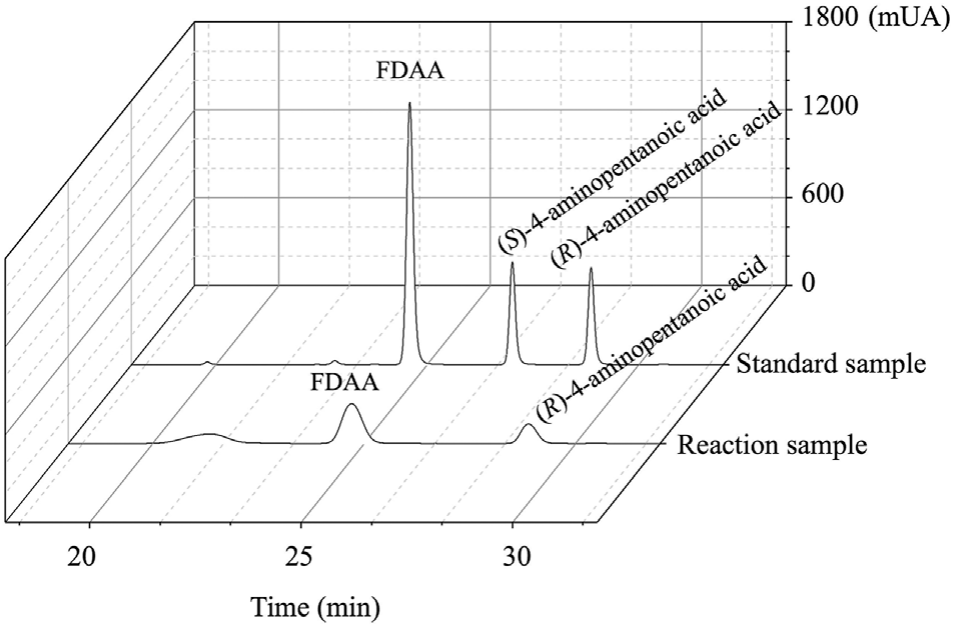
The HPLC spectrum from FDAA derivatization of the product synthesized by *Ec*GDH^K116Q/N348M^ and commercial racemic 4-aminopentanoic acid.

**FIGURE 7:**
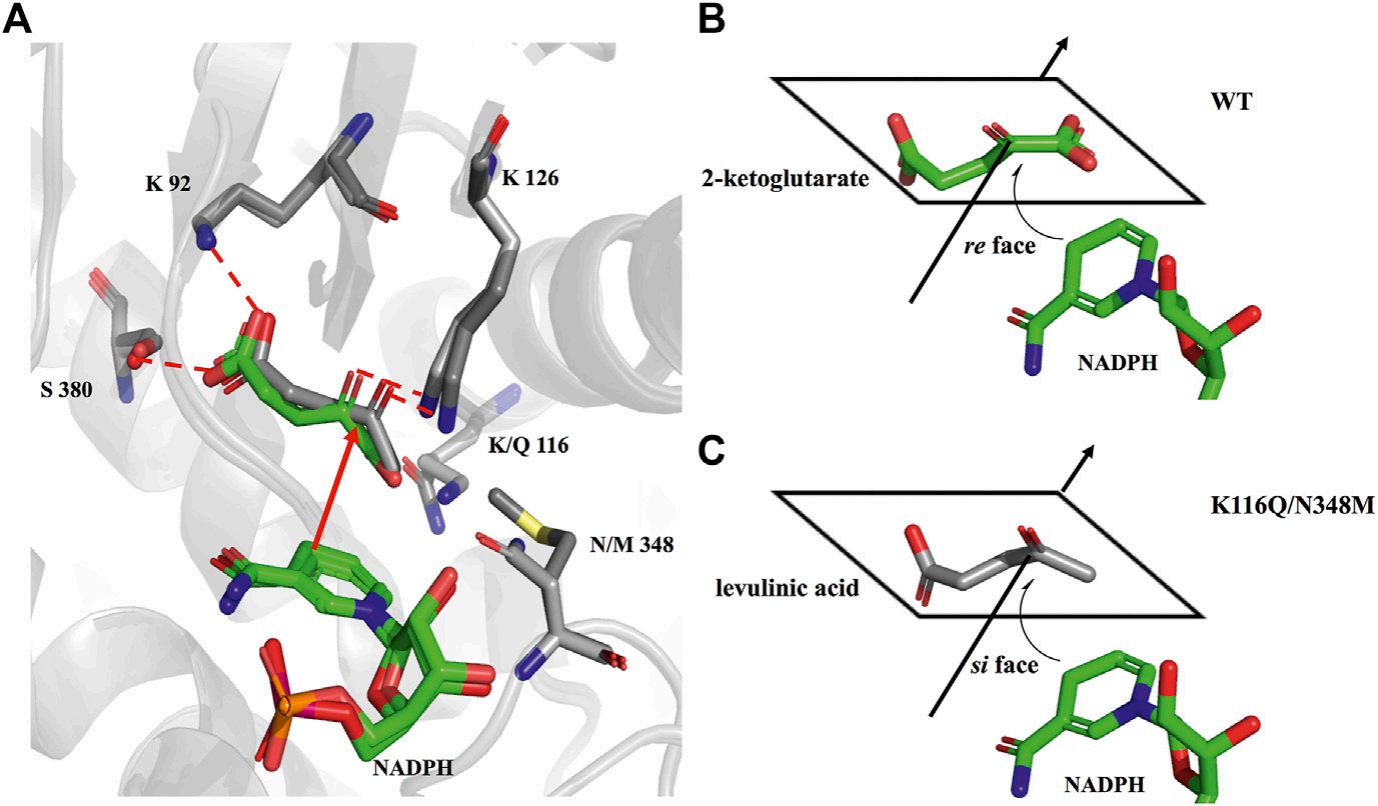
Comparison of the docking results. **(A)** Structural alignment analysis of docking pose for 2-ketoglutarate in the catalytic pocket of wild-type and LA in the catalytic pocket of *Ec*GDH^K116Q/N348M^. **(B)** schematic representation of NADPH attacking 2-ketoglutarate. **(C)** schematic representation of NADPH attacking LA.

As shown in Figure 6, the HPLC analysis of the reaction solution catalyzed by *Ec*GDH^K116Q/N348M^ revealed that the reaction product was (*R*)-4-aminopentanoic acid, which is different from the product configuration catalyzed by the natural amine dehydrogenase (Mayol et al., 2016). To clarify the reasons for the configuration of this specific product, the docking results and catalytic mechanism of GDH were analyzed. As shown in Figure 7A, two residues (K92 and S380 of the wild-type or *Ec*GDH^K116Q/N348M^) formed hydrogen bones with the γ-carboxyl group of 2-ketoglutarate or the carboxyl group of LA, and the substrate carbonyl group was stabilized by the side chain of K126 before a hydride was supplied by the NADPH to the carbonyl carbon atom (Tomita et al., 2017). Moreover, the coenzyme NADPH attacked the 2-ketoglutarate from the *re* face (Figure 7B). However, when the substrate changed from 2-ketoglutarate to LA, the NADPH attacked the LA from the *si* face, which determines the stereochemistry of the product (*R*)-4-aminopentanoic acid (Figure 7C). These observations are consistent with the HPLC analysis of the reaction solution. The natural amine dehydrogenase was obtained from the NCBI database using the protein sequence of (2*R*,4*S*)-2,4-diaminopentanoate dehydrogenase from *Clostridium sticklandii* (2,4-DAPDH, EC 1.4.1.12), which can catalyze (2*R*)-2-amino-4-oxopentanoate to (2*R*,4*S*)-2,4-diaminopentanoate, as a template (Fukuyama et al., 2014; Mayol et al., 2016). From the perspective of the catalytic mechanism, 2,4-DAPDH and *Ec*GDH (EC 1.4.1.4) have opposite coenzyme offensive surfaces, which results in different product stereoselectivities (Xue et al., 2018).

### Kinetic Parameters and Substrate Scope

To compare the catalytic efficiency of the *Ec*GDH^K116C^ and the best mutant *Ec*GDH^K116Q/N348M^, kinetic parameters for LA and NADPH were determined (Table 1). The *k*_cat_/*K*_m_ of *Ec*GDH^K116Q/N348M^ for LA and NADPH were 42.0- and 7.9-fold higher, respectively, than that of *Ec*GDH^K116C^. And *Ec*GDH^K116Q/N348M^ had a higher affinity and *k*_cat_ for LA than *Ec*GDH^K116C^. This may be because N348 has a negative effect on the binding of the enzyme and LA, thereby reducing the probability of LA to form the correct pose in the pocket. However, *Ec*GDH^K116C^ had higher coenzyme affinity than the *Ec*GDH^K116Q/N348M^. According to the crystal structure of 5ijz, N347 (corresponding to N348 in *Ec*GDH) can form hydrogen bonds with the coenzyme and stabilize it (Son et al., 2015). Therefore, the mutation of *Ec*GDH^K116Q/N348M^ at site 348 makes its affinity for NADPH weaker than that of *Ec*GDH^K116C^. The *K*_m_ and *k*_cat_ of *Ec*GDH^K116Q/N348M^ to LA were very different from those of the wild-type GDH for 2-ketoglutarate (Sharkey and Engel, 2009), which indicates that the two-point mutations at residues 116 and 348 weaken the enzyme-substrate interaction, leading to a decrease in *k*_cat_ and affinity.

**TABLE 1:** Kinetic data for the asymmetric amination of LA with *Ec*GDH^K116C^ and *Ec*GDH^K116Q/N348M^.

Substrate	Enzyme	*V*_max_ (U/mg)	*K*_m_ (mM)	*k*_cat_ (s^−1^)	*k*_cat_/*K*_m_ (mM^−1^ s^−1^)
LA^a^	*Ec*GDH^K116C^	0.109	1,340 ± 24.3	0.088 ± 0.0013	6.6 × 10^–5^
	*Ec*GDH^K116Q/N348M^	2.82	824.0 ± 13.6	2.28 ± 0.19	2.77 × 10^–3^
NADPH^b^	*Ec*GDH^K116C^	0.0834	0.0988 ± 0.003	0.067 ± 0.0037	0.68
	*Ec*GDH^K116Q/N348M^	1.87	0.28 ± 0.07	1.51 ± 0.23	5.39

a The mixture composed of Tris-HCl (100 mM, pH 8.5), NH_4_Cl-NH_4_OH (0.8 M, pH 8.5), 0.2 mM NADPH, and different concentrations of LA (0–1,600 mM) was incubated at 30°C for 1 min before adding purified enzyme.

b The mixture containing Tris-HCl (100 mM, pH 8.5), NH_4_Cl-NH_4_OH (0.8 M, pH 8.5) different concentrations of NADPH (0–0.8 mM), and 80 mM LA was incubated at 30°C for 1 min before adding purified enzyme.

The substrate specificity of the wild-type and mutants (K116C, and K116Q/N348M) were profiled by activity assays over a group of structurally diverse carbonyl compounds. The mutants displayed no activity toward fatty ketones (2-butanone, 2-pentanone, and 4-methyl-2-pentanone), aromatic ketones (acetophenone, phenylacetone, and phenylbutanone) and alcohol ketones (4-hydroxy-2-butanone, and 5-hydroxy-2-pentanone). Therefore, γ-carbonyl acid and its derivatives, which are more similar to the natural substrate 2-ketoglutarate, were used as substrates in this analysis. As shown in Table 2, the activity of *Ec*GDH^K116Q/N348M^ on natural substrates was lower than that of the wild-type, but the substrate spectrum of *Ec*GDH^K116Q/N348M^ was expanded compared with that of the wild-type and *Ec*GDH^K116C^. Since the activity of *Ec*GDH^K116Q/N348M^ on non-natural substrates was generally low, further evolution of this engineered enzyme is required to improve its activity.

**TABLE 2:** Activity of wild type, *Ec*GDH^K116C^ and *Ec*GDH^K116Q/N348M^ towards various γ-carbonyl acid and its derivatives^a^.

Substrate	Structural formula	*Ec*GDH (U/mg)	*Ec*GDH^K116C^ (mU/mg)	*Ec*GDH^K116Q/N348M^ (mU/mg)
S1	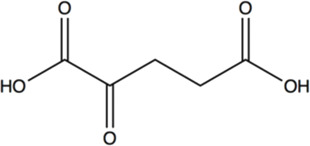	156.4 ± 11.4	21.8 ± 2.6	294.2 ± 51.0
S2	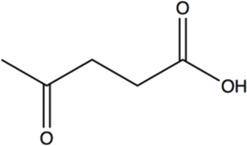	N.A.^b^	10.1 ± 0.2	108.6 ± 1.5
S3	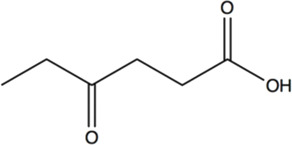	N.A.	N.A.	10.3 ± 0.7
S4	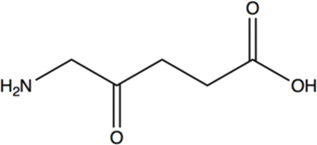	N.A.	N.A.	19.5 ± 3.7

a Each value was calculated from three independent experiments. The mixture composed of Tris-HCl (100 mM, pH 8.5), NH_4_Cl-NH_4_OH (0.8 M, pH 8.5), 0.2 mM NADPH, and 80 mM substrate was incubated at 30°C for 1 min before adding purified enzyme. Optimization of conditions for specific activity determination see Supplementary Figure S2.

b N.A. = No measurable activity.

### Conversion With the Cofactor Recycle System

**FIGURE 8:**
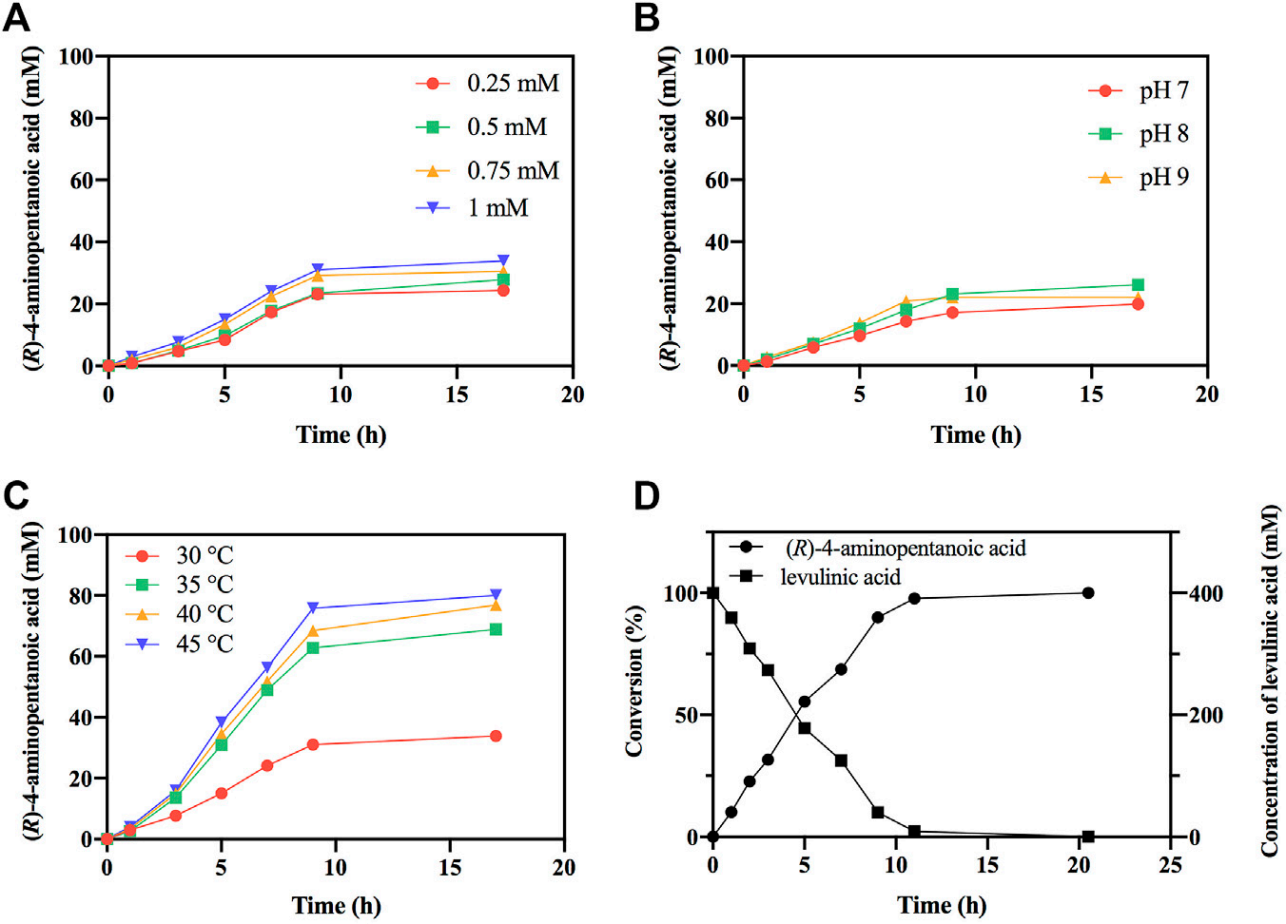
Optimization of reaction conditions. **(A)** The effect of NADP^+^ concentration on reductive amination reaction by *Ec*GDH^K116Q/N348M^. **(B)** The effect of pH on reductive amination reaction by *Ec*GDH^K116Q/N348M^. **(C)** The effect of temperature on reductive amination reaction by *Ec*GDH^K116Q/N348M^. **(D)** Time courses of reductive amination of LA by *Ec*GDH^K116Q/N348M^ under the optimal conditions. Conversions determined by HPLC analysis.

Subsequently, the reductive amination reactions of LA by *Ec*GDH^K116Q/N348M^ coupled with *Bs*FDH for regeneration of NADPH were performed under different conditions (different NADP^+^ concentration, pH and temperature). As shown in Figures 8A,C, as the concentration of the coenzyme and the temperature increased, the conversion efficiency also increased, and the reaction showed the best conversion efficiency at pH 8 (Figure 8B). Under optimal conditions (1 mM NADP^+^, pH 8 and 45°C), *Ec*GDH^K116Q/N348M^ converted 0.4 M LA to (*R*)-4-aminopentanoic acid (>99% *ee*) by more than 97% in 11 h with the addition of 1.51 mg/ml *Ec*GDH and 0.20 mg/ml *Bs*FDH (Figure 8D). Comparisons with other studies (Table 3) revealed that the approach for (*R*)-4-aminopentanoic acid production in this study had significant advantages in reaction conditions, conversion efficiency and product optical purity.

**TABLE 3:** Comparison of (*R*)-4-aminopentanoic acid synthesis results reported in the literature^a^.

Entry	Reaction substrate	Reaction conditions	Time	Conversion rates (%)	Optical purity	Ref
1	α,β-unsaturated γ-amino ester (4.5 mmol)	HCONH_4_/Pd (H_2_; MeOH, heating)	—	81	72% *ee*	Palacios et al. (2001)
2	LA (8 mmol) + FA + ammonia	Au/ZrO_2_-VS (N_2_, 5 atm; 130°C)	16 h	90	Racemic	Du et al. (2011)
3	LA (2 mmol) + FAM	FA (160°C)	1.5 h	>93	Racemic	Wu et al. (2019)
4	LA (1 mmol) + NH_3_ (gas, 0.5 Mpa)	Pt/P-TiO_2_ (H_2_, 1.5 Mpa; MeOH)	72 h	>89	Racemic	Xie et al. (2019)
5	LA (0.01–0.05 mmol) + (*R*)-α-MBA	(*R*)-AT (30°C; 160 rpm)	12–16 h	—	>99% *ee*	Jiang et al. (2015)
6	LA (4 mmol) + AF + NADP^+^	*Ec*GDH^K116Q/N348M^, *Bs*FDH (45°C; 200 rpm)	11 h	>97	>99% *ee*	This study

a Abbreviations used: FA, Formic acid; FAM, formamide; MeOH, Methyl alcohol; (*R*)-AT, (*R*)-selective amine transaminase; (*R*)-α-MBA, (*R*)-1-methylbenzylamine; AF, Ammonium formate.

## Conclusion

The previous research in engineered amine dehydrogenases gained from leucine dehydrogenase or phenylalanine dehydrogenase (Abrahamson et al., 2012; Abrahamson et al., 2013; Ye et al., 2015; Chen et al., 2018) greatly solves the problem of the lack of natural amine dehydrogenase (Mayol et al., 2016; Bommarius, 2019). However, the substrate specificity of the engineered amine dehydrogenase is limited to the synthesis of aliphatic and aromatic chiral amines. (*R*)-selective amine transaminases can catalyze the synthesis of (*R*)-4-aminopentanoic acid with high optical purity; however, the amination catalysed by (*R*)-selective amine transaminases, using (*R*)-1-methylbenzylamine as an amine donor, requires removal of the co-product acetophenone to shift the unfavorable thermodynamic equilibrium. In this study, the engineered GDH provides a new way for the synthesis of γ-amino acids and enriches the toolbox of amine dehydrogenases. Coupled with *Bs*FDH, *Ec*GDH^K116Q/N348M^ converted 0.4 M LA to (*R*)-4-aminopentanoic acid by more than 97% in 11 h with excellent stereoselectivity (>99% *ee*). Moreover, the reaction system can use cheap ammonia as amino donor and generate only inorganic carbonate as byproduct, and the substrate LA in the reaction is one of the top 12 carbohydrate-derived compounds listed by Department of Energy United States that can be obtained from the lignocellulosic biomass (Weingarten et al., 2012; Pileidis and Titirici, 2016; Girisuta and Heeres, 2017; Habe et al., 2020; Wang et al., 2020); the product (*R*)-4-aminopentanoic acid is an important intermediate for the synthesis of psychotropic drugs (Trotter et al., 2005) and muscarinic M4 receptor agonist (Brown et al., 2018), and it can also participate in the formation of physiologically active artificial peptides (Grison et al., 2016). Taken together, these results indicated that this pathway can sustainably synthesize high value-added (*R*)-4-aminopentanoic acid.

## Data Availability

The datasets presented in this study can be found in online repositories. The names of the repository/repositories and accession number(s) can be found in the article/Supplementary Material.

## References

[B1] Abrahamson M. J. Vázquez-Figueroa E. Woodall N. B. Moore J. C. Bommarius A. S. (2012). Development of an Amine Dehydrogenase for Synthesis of Chiral Amines. Angew. Chem. Int. Ed. 51, 3969–3972. 10.1002/anie.201107813 22396126

[B2] Abrahamson M. J. Wong J. W. Bommarius A. S. (2013). The Evolution of an Amine Dehydrogenase Biocatalyst for the Asymmetric Production of Chiral Amines. Adv. Synth. Catal. 355, 1780–1786. 10.1002/adsc.201201030

[B3] Bhushan R. Bruckner H. (2004). Marfey?s Reagent for Chiral Amino Acid Analysis: A Review. Amino Acids 27, 231–247. 10.1007/s00726-004-0118-0 15503232

[B4] Bommarius A. S. (2019). Amine Dehydrogenases Occur in Nature. Nat. Catal. 2, 288–289. 10.1038/s41929-019-0270-2

[B5] Brown G. A. Congreve M. S. Pickworth M. Rackham M. Tehan B. G. (2018). Muscarinic Agonists. U.S. Patent No US 20180228791 A1 U.S. Patent and Trademark Office Washington, DC.

[B6] Cai R.-F. Liu L. Chen F.-F. Li A. Xu J.-H. Zheng G.-W. (2020). Reductive Amination of Biobased Levulinic Acid to Unnatural Chiral γ-Amino Acid Using an Engineered Amine Dehydrogenase. ACS Sustain. Chem. Eng. 8, 17054–17061. 10.1021/acssuschemeng.0c04647

[B7] Chen F.-F. Zheng G.-W. Liu L. Li H. Chen Q. Li F.-L. (2018). Reshaping the Active Pocket of Amine Dehydrogenases for Asymmetric Synthesis of Bulky Aliphatic Amines. ACS Catal. 8, 2622–2628. 10.1021/acscatal.7b04135

[B8] Chen Q.-Y. Liu Y. Cai W. Luesch H. (2014). Improved Total Synthesis and Biological Evaluation of Potent Apratoxin S4 Based Anticancer Agents with Differential Stability and Further Enhanced Activity. J. Med. Chem. 57, 3011–3029. 10.1021/jm4019965 24660812 PMC3993931

[B9] Comegna D. de Paola I. Saviano M. Del Gatto A. Zaccaro L. (2015). Straightforward Entry to *S*-Glycosylated Fmoc-Amino Acids and Their Application to Solid Phase Synthesis of Glycopeptides and Glycopeptidomimetics. Org. Lett. 17, 640–643. 10.1021/ol503664t 25622618

[B10] Du X.-L. He L. Zhao S. Liu Y.-M. Cao Y. He H.-Y. (2011). Hydrogen-Independent Reductive Transformation of Carbohydrate Biomass into γ-Valerolactone and Pyrrolidone Derivatives with Supported Gold Catalysts. Angew. Chem. Int. Ed. 50, 7815–7819. 10.1002/anie.201100102 21732502

[B11] Fukuyama S. Mihara H. Miyake R. Ueda M. Esaki N. Kurihara T. (2014). Characterization of a Thermostable 2,4-diaminopentanoate Dehydrogenase from *Fervidobacterium Nodosum* Rt17-B1. J. Biosci. Bioeng. 117, 551–556. 10.1016/j.jbiosc.2013.11.002 24326351

[B12] Girisuta B. Heeres H. J. (2017). “Levulinic Acid from Biomass: Synthesis and Applications,” in “Levulinic Acid from Biomass: Synthesis and Applications,” in *Production Of Platform Chemicals from Sustainable Resources* . Editors Fang Z. Smith R. Qi X. (Singapore: Springer), 143–169. 10.1007/978-981-10-4172-3_5

[B13] Gómez J. E. Guo W. Gaspa S. Kleij A. W. (2017). Copper-Catalyzed Synthesis of γ-Amino Acids Featuring Quaternary Stereocenters. Angew. Chem. Int. Ed. 56, 15035–15038. 10.1002/anie.201709511 29024315

[B14] Grison C. M. Miles J. A. Robin S. Wilson A. J. Aitken D. J. (2016). An α-Helix-Mimicking 12,13-Helix: Designed α/β/γ-Foldamers as Selective Inhibitors of Protein-Protein Interactions. Angew. Chem. Int. Ed. 55, 11096–11100. 10.1002/anie.201604517 PMC5014220 27467859

[B15] Habe H. Sato Y. Kirimura K. (2020). Microbial and Enzymatic Conversion of Levulinic Acid, an Alternative Building Block to Fermentable Sugars from Cellulosic Biomass. Appl. Microbiol. Biotechnol. 104, 7767–7775. 10.1007/s00253-020-10813-7 32770274

[B16] Hanson R. L. Davis B. L. Goldberg S. L. Johnston R. M. Parker W. L. Tully T. P. (2008). Enzymatic Preparation of a D-Amino Acid from a Racemic Amino Acid or Keto Acid. Org. Process. Res. Dev. 12, 1119–1129. 10.1021/op800149q

[B17] Jiang J. Chen X. Zhang D. Wu Q. Zhu D. (2015). Characterization of (*R*)-selective Amine Transaminases Identified by In Silico Motif Sequence Blast. Appl. Microbiol. Biotechnol. 99, 2613–2621. 10.1007/s00253-014-6056-1 25267157

[B18] Kato Y. Fusetani N. Matsunaga S. Hashimoto K. Fujita S. Furuya T. (1986). Bioactive marine Metabolites. Part 16. Calyculin A. A Novel Antitumor Metabolite from the marine Sponge Discodermia Calyx. J. Am. Chem. Soc. 108, 2780–2781. 10.1021/ja00270a061

[B19] Knaus T. Böhmer W. Mutti F. G. (2017). Amine Dehydrogenases: Efficient Biocatalysts for the Reductive Amination of Carbonyl Compounds. Green. Chem. 19, 453–463. 10.1039/C6GC01987K 28663713 PMC5486444

[B20] Li J. Pan J. Zhang J. Xu J.-H. (2014). Stereoselective Synthesis of L-*Tert*-Leucine by a Newly Cloned Leucine Dehydrogenase from *Exiguobacterium Sibiricum* . J. Mol. Catal. B: Enzymatic 105, 11–17. 10.1016/j.molcatb.2014.03.010

[B21] Mayol O. David S. Darii E. Debard A. Mariage A. Pellouin V. (2016). Asymmetric Reductive Amination by a Wild-type Amine Dehydrogenase from the Thermophilic Bacteria *Petrotoga Mobilis* . Catal. Sci. Technol. 6, 7421–7428. 10.1039/C6CY01625A

[B22] Palacios F. Aparicio D. García J. Rodríguez E. Fernández-Acebes A. (2001). A Convenient Synthesis of Racemic and Optically Active 1-Aza-1,3-Dienes Derived from γ-amino Esters: Reduction to α,β-unsaturated and Saturated γ-amino Acid Derivatives. Tetrahedron 57, 3131–3141. 10.1016/S0040-4020(01)00171-5

[B23] Patil M. D. Grogan G. Bommarius A. Yun H. (2018). Oxidoreductase-catalyzed Synthesis of Chiral Amines. ACS Catal. 8, 10985–11015. 10.1021/acscatal.8b02924

[B24] Patrick W. M. Firth A. E. (2005). Strategies and Computational Tools for Improving Randomized Protein Libraries. Biomol. Eng. 22, 105–112. 10.1016/j.bioeng.2005.06.001 16095966

[B25] Pettit G. R. Kamano Y. Herald C. L. Tuinman A. A. Boettner F. E. Kizu H. (1987). The Isolation and Structure of a Remarkable marine Animal Antineoplastic Constituent: Dolastatin 10. J. Am. Chem. Soc. 109, 6883–6885. 10.1021/ja00256a070

[B26] Pileidis F. D. Titirici M.-M. (2016). Levulinic Acid Biorefineries: New Challenges for Efficient Utilization of Biomass. ChemSusChem 9, 562–582. 10.1002/cssc.201501405 26847212

[B27] Reetz M. T. Kahakeaw D. Lohmer R. (2008). Addressing the Numbers Problem in Directed Evolution. Chembiochem 9, 1797–1804. 10.1002/cbic.200800298 18567049

[B28] Robert X. Gouet P. (2014). Deciphering Key Features in Protein Structures with the New ENDscript Server. Nucleic Acids Res. 42, W320–W324. 10.1093/nar/gku316 24753421 PMC4086106

[B29] Schägger H. (2006). Tricine-SDS-PAGE. Nat. Protoc. 1, 16–22. 10.1038/nprot.2006.4 17406207

[B30] Schwede T. Kopp J. Guex N. Peitsch M. C. (2003). SWISS-MODEL: An Automated Protein Homology-Modeling Server. Nucleic Acids Res. 31, 3381–3385. 10.1093/nar/gkg520 12824332 PMC168927

[B31] Sharkey M. A. Engel P. C. (2009). Modular Coenzyme Specificity: a Domain-Swopped Chimera of Glutamate Dehydrogenase. Proteins 77, 268–278. 10.1002/prot.22433 19425107

[B32] Sievers F. Wilm A. Dineen D. Gibson T. J. Karplus K. Li W. (2011). Fast, Scalable Generation of High‐quality Protein Multiple Sequence Alignments Using Clustal Omega. Mol. Syst. Biol. 7, 539. 10.1038/msb.2011.75 21988835 PMC3261699

[B33] Son H. F. Kim I.-K. Kim K.-J. (2015). Structural Insights into Domain Movement and Cofactor Specificity of Glutamate Dehydrogenase from *Corynebacterium Glutamicum* . Biochem. Biophysical Res. Commun. 459, 387–392. 10.1016/j.bbrc.2015.02.109 25727019

[B34] Stillman T. J. Baker P. J. Britton K. L. Rice D. W. (1993). Conformational Flexibility in Glutamate Dehydrogenase. J. Mol. Biol. 234, 1131–1139. 10.1006/jmbi.1993.1665 8263917

[B35] Stratmann K. Burgoyne D. L. Moore R. E. Patterson G. M. L. Smith C. D. (1994). Hapalosin, a Cyanobacterial Cyclic Depsipeptide with Multidrug-Resistance Reversing Activity. J. Org. Chem. 59, 7219–7226. 10.1021/jo00103a011

[B36] Tang L. Gao H. Zhu X. Wang X. Zhou M. Jiang R. (2012). Construction of "Small-Intelligent" Focused Mutagenesis Libraries Using Well-Designed Combinatorial Degenerate Primers. BioTechniques 52, 149–158. 10.2144/000113820 22401547

[B37] Tomita T. Yin L. Nakamura S. Kosono S. Kuzuyama T. Nishiyama M. (2017). Crystal Structure of the 2-Iminoglutarate-Bound Complex of Glutamate Dehydrogenase fromCorynebacterium Glutamicum. FEBS Lett. 591, 1611–1622. 10.1002/1873-3468.12667 28486765

[B38] Trotter N. S. Brimble M. A. Harris P. W. R. Callis D. J. Sieg F. (2005). Synthesis and Neuroprotective Activity of Analogues of Glycyl-L-Prolyl-L-Glutamic Acid (GPE) Modified at the α-carboxylic Acid. Bioorg. Med. Chem. 13, 501–517. 10.1016/j.bmc.2004.10.005 15598572

[B39] Vasudev P. G. Chatterjee S. Shamala N. Balaram P. (2011). Structural Chemistry of Peptides Containing Backbone Expanded Amino Acid Residues: Conformational Features of β, γ, and Hybrid Peptides. Chem. Rev. 111, 657–687. 10.1021/cr100100x 20843067

[B40] Wang J. Cui H. Wang Y. Zhao R. Xie Y. Wang M. (2020). Efficient Catalytic Conversion of Cellulose to Levulinic Acid in the Biphasic System of Molten Salt Hydrate and Methyl Isobutyl Ketone. Green. Chem. 22, 4240–4251. 10.1039/D0GC00897D

[B41] Weingarten R. Conner W. C. Huber G. W. (2012). Production of Levulinic Acid from Cellulose by Hydrothermal Decomposition Combined with Aqueous Phase Dehydration with a Solid Acid Catalyst. Energy Environ. Sci. 5, 7559–7574. 10.1039/C2EE21593D

[B42] Wu H. Dai W. Saravanamurugan S. Li H. Yang S. (2019). Quasi-catalytic Approach to N-Unprotected Lactams via Transfer Hydro-Amination/cyclization of Biobased Keto Acids. ACS Sustain. Chem. Eng. 7, 10207–10213. 10.1021/acssuschemeng.9b00412

[B43] Xie C. Song J. Wu H. Hu Y. Liu H. Zhang Z. (2019). Ambient Reductive Amination of Levulinic Acid to Pyrrolidones over Pt Nanocatalysts on Porous TiO2 Nanosheets. J. Am. Chem. Soc. 141, 4002–4009. 10.1021/jacs.8b13024 30739440

[B44] Xue Y.-P. Cao C.-H. Zheng Y.-G. (2018). Enzymatic Asymmetric Synthesis of Chiral Amino Acids. Chem. Soc. Rev. 47, 1516–1561. 10.1039/c7cs00253j 29362736

[B45] Ye L. J. Toh H. H. Yang Y. Adams J. P. Snajdrova R. Li Z. (2015). Engineering of Amine Dehydrogenase for Asymmetric Reductive Amination of Ketone by Evolving *Rhodococcus* Phenylalanine Dehydrogenase. ACS Catal. 5, 1119–1122. 10.1021/cs501906r

[B46] Zhang D. Jing X. Zhang W. Nie Y. Xu Y. (2019). Highly Selective Synthesis of D-Amino Acids from Readily Available L-Amino Acids by a One-Pot Biocatalytic Stereoinversion cascade. RSC Adv. 9, 29927–29935. 10.1039/C9RA06301C PMC9072125 35531513

