# Supplementary material for: A Sustainable Approach for Synthesizing (R)-4-Aminopentanoic Acid From Levulinic Acid Catalyzed by Structure-Guided Tailored Glutamate Dehydrogenase
Source: Front Bioeng Biotechnol. 2022 Jan 10;9:770302. doi: 10.3389/fbioe.2021.770302 (PMC8784811; doi:10.3389/fbioe.2021.770302)
Supplement: Supplementary file 1 [file DataSheet1.docx]

Supplementary Material

**Supplementary Tables**

**Table S1. Primers used in the research**

| Primers | Primer sequences 5’-3’ |
| --- | --- |
| *Ec*GDH-K116F-F | GGCTTTGAACAAACCTTCTTCAATGCCCTGACTACTCTGCCG |
| *Ec*GDH-K116L-F | GGCTTTGAACAAACCTTCCTGAATGCCCTGACTACTCTGCCG |
| *Ec*GDH-K116I-F | GGCTTTGAACAAACCTTCATCAATGCCCTGACTACTCTGCCG |
| *Ec*GDH-K116M-F | GGCTTTGAACAAACCTTCATGAATGCCCTGACTACTCTGCCG |
| *Ec*GDH-K116V-F | GGCTTTGAACAAACCTTCGTTAATGCCCTGACTACTCTGCCG |
| *Ec*GDH-K116P-F | GGCTTTGAACAAACCTTCCCGAATGCCCTGACTACTCTGCCG |
| *Ec*GDH-K116T-F | GGCTTTGAACAAACCTTCACCAATGCCCTGACTACTCTGCCG |
| *Ec*GDH-K116A-F | GGCTTTGAACAAACCTTCGCGAATGCCCTGACTACTCTGCCG |
| *Ec*GDH-K116Y-F | GGCTTTGAACAAACCTTCTACAATGCCCTGACTACTCTGCCG |
| *Ec*GDH-K116H-F | GGCTTTGAACAAACCTTCCACAATGCCCTGACTACTCTGCCG |
| *Ec*GDH-K116Q-F | GGCTTTGAACAAACCTTCCAGAATGCCCTGACTACTCTGCCG |
| *Ec*GDH-K116D-F | GGCTTTGAACAAACCTTCGACAATGCCCTGACTACTCTGCCG |
| *Ec*GDH-K116E-F | GGCTTTGAACAAACCTTCGAAAATGCCCTGACTACTCTGCCG |
| *Ec*GDH-K116C-F | GGCTTTGAACAAACCTTCTGCAATGCCCTGACTACTCTGCCG |
| *Ec*GDH-K116W-F | GGCTTTGAACAAACCTTCTGGAATGCCCTGACTACTCTGCCG |
| *Ec*GDH-K116R-F | GGCTTTGAACAAACCTTCCGTAATGCCCTGACTACTCTGCCG |
| *Ec*GDH-K116G-F | GGCTTTGAACAAACCTTCGGTAATGCCCTGACTACTCTGCCG |
| *Ec*GDH-K116N-F | GGCTTTGAACAAACCTTCAACAATGCCCTGACTACTCTGCCG |
| *Ec*GDH-K116S-F | GGCTTTGAACAAACCTTCTCTAATGCCCTGACTACTCTGCCG |
| *Ec*GDH-K116-F | GGCTTTGAACAAACCTTCAAAAATGCCCTGACTACTCTGCCG |
| V-*Ec*GDH-116-R | GAAGGTTTGTTCAAAGCCGAGG |
| V-*Ec*GDH-348-F | ATGCCGACCACCATCGAAGC |
| *Ec*GDH-348AHN-R | GATGGTGGTCGGCATAHNTGCCCCTTCGGCGAC |
| *Ec*GDH-348TKB-R | GATGGTGGTCGGCATTKBTGCCCCTTCGGCGAC |
| *Ec*GDH-348CAT-R | GATGGTGGTCGGCATCATTGCCCCTTCGGCGAC |
| *Ec*GDH-348CCA-R | GATGGTGGTCGGCATCCATGCCCCTTCGGCGAC |

The underline indicates the homologous region, and the red indicates the mutation site.

**Table S2. Specific activity of obtained mutants toward levulinic acid**

| Mutations | specific activity (mU/mg) |
| --- | --- |
| *Ec*GDH-K116A | 8.2±2.7 |
| *Ec*GDH-K116C | 10.1±0.2 |
| *Ec*GDH-K116D | 0 |
| *Ec*GDH-K116E | 0 |
| *Ec*GDH-K116F | 0 |
| *Ec*GDH-K116G | 0 |
| *Ec*GDH-K116H | 0 |
| *Ec*GDH-K116I | 0 |
| *Ec*GDH-K116 | 0 |
| *Ec*GDH-K116L | 0 |
| *Ec*GDH-K116M | 3.2±0.3 |
| *Ec*GDH-K116N | 0 |
| *Ec*GDH-K116P | 0 |
| *Ec*GDH-K116Q | 4.8±0.3 |
| *Ec*GDH-K116R | 7.5±0.1 |
| *Ec*GDH-K116S | 6.2±0.4 |
| *Ec*GDH-K116T | 2.2±0.4 |
| *Ec*GDH-K116V | 0 |
| *Ec*GDH-K116W | 0 |
| *Ec*GDH-K116Y | 0 |
| *Ec*GDH-K116M/N348T | 11.5±2.1 |
| *Ec*GDH-K116E/N348M | 33.7±7.6 |
| *Ec*GDH-K116S/N348L | 50.1±7.6 |
| *Ec*GDH-K116Q/N348M | 108.6±1.5 |

**Supplementary Figures**

**

**

**Fig. S1. Construction method of two-site combinatorial saturation mutagenesis library.**

**

**

**Fig. S2. Optimum pH, temperature and** **stability of** **GDH^K116Q/N348M^ using levulinic acid as substrate.**

(A) The pH stability: enzyme inactivation assay at different pH (6.0-10.0) for 14 h;

(B) Optimum pH: the enzyme activity was assayed at various pH (7.0-10.0) in 0.1 M Tris-HCl buffer;

(C) Thermal stability analysis of GDH^K116Q/N348M^ was performed in 0.1 M Tris-HCl buffer (pH 7.5) at 40 °C (The half-life of GDH^K116Q/N348M^ at 40 °C is 8.9 h) ^a^;

(D) Temperature optima: the enzyme activity was measured at various temperatures (30-70 °C) in 0.1 M Tris-HCl buffer (pH 8.5).

^a^ The value of t_1/2_ at 40 °C was calculated using the following formula: t_1/2_=ln2 k^-1^ (k is first-order rate constant which is deduced from the semilogarithmic plot of incubation time *versus* residual activity). (Le et al., 2012)

**Reference**

Le, Q. A. T., Joo, J. C., Yoo, Y. J., and Kim, Y. H. (2012). Development of thermostable Candida antarctica lipase B through novel in silico design of disulfide bridge. *Biotechnol. Bioeng.* 109, 867–876. doi:10.1002/bit.24371.
